# Day-Zero Serum FTIR Spectroscopy Identifies a Biochemical Signature Associated with Functional Pancreas Graft Dysfunction After Simultaneous Pancreas–Kidney Transplantation

**DOI:** 10.3390/life16071054

**Published:** 2026-06-24

**Authors:** Emanuel Vigia, Luís Ramalhete, Rúben Araújo, Sofia Corado, Inês Barros, Beatriz Chumbinho, Ana Nobre, Sofia Carrelha, Paula Pico, Fernando Rodrigues, Miguel Bigotte, Rita Magriço, Patrícia Cotovio, Fernando Caeiro, Inês Aires, Cecília Silva, Ana Pena, Luís Bicho, Cristina Jorge, Cecília R. C. Calado, Jorge P. Pereira, Aníbal Ferreira, Hugo P. Marques

**Affiliations:** 1 Hepatobiliopancreatic and Transplantation Centre, Curry Cabral Hospital, Unidade Local De Saúde De São José, R. Da Beneficência 8, 1050-099 Lisbon, Portugal; sofiacorado@gmail.com (S.C.); inesfigueiredodebarros@gmail.com (I.B.); beatriz.chumbinho@gmail.com (B.C.); nobre.ana73@gmail.com (A.N.); sofia_carrelha@hotmail.com (S.C.); anapena64@gmail.com (A.P.); bicho.luis@gmail.com (L.B.); hugoscpm@gmail.com (H.P.M.); 2 Nova Medical School, Faculdade de Ciências Médicas, NMS, FCM, Universidade Nova de Lisboa, 1169-056 Lisbon, Portugal; luis.m.ramalhete@gmail.com (L.R.); rubenalexandredinisaraujo@gmail.com (R.A.); mbigottevieira@gmail.com (M.B.); rita.remedios@ulssjose.min-saude.pt (R.M.); patriciacotovio@gmail.com (P.C.); ircaires@gmail.com (I.A.); cecilia.silva77@gmail.com (C.S.); fusilis@gmail.com (J.P.P.); anibalferreira@netcabo.pt (A.F.); 3 INOVA4Health-Advancing Precision Medicine, Núcleo de Investigação em Doenças Renais, Nova Medical School, Faculdade de Ciências Médicas, NMS, FCM, Universidade Nova de Lisboa, 1169-056 Lisbon, Portugal; 4 Centro Clínico Académico de Lisboa, 1169-024 Lisbon, Portugal; 5 Blood and Transplantation Centre of Lisbon, Instituto Português do Sangue e da Transplantação, Alameda das Linhas de Torres, n.º 117, 1769-001 Lisbon, Portugal; 6 ISEL-Instituto Superior de Engenharia de Lisboa, Rua Conselheiro Emídio Navarro 1, 1959-007 Lisbon, Portugal; cecilia.calado@isel.pt; 7 Gabinete Coordenador de Colheita e Transplantação, Hospital São José, Unidade Local de Saúde de São José R. José António Serrano, 1150-199 Lisboa, Portugal; paulac.pico@chlc.min-saude.pt (P.P.); fernando.rodrigues@ulssjose.min-saude.pt (F.R.); 8 Nephrology, Hospital Curry Cabral, Unidade Local De Saúde De São José, R. da Beneficência 8, 1050-099 Lisbon, Portugal; cristina.jorge2@ulssjose.min-saude.pt; 9 Institute for Bioengineering and Biosciences (IBB), The Associate Laboratory Institute for Health and Bioeconomy-I4HB, Instituto Superior Técnico (IST), Universidade de Lisboa (UL), Av. Rovisco Pais, 1049-001 Lisbon, Portugal

**Keywords:** simultaneous pancreas–kidney transplantation, pancreas graft dysfunction, loss of insulin independence, FTIR spectroscopy, serum biomarker, machine learning, Naïve Bayes, FCBF, leave-one-out cross-validation, spectral quality control, risk stratification

## Abstract

Background: Simultaneous pancreas–kidney (SPK) transplantation can restore renal function and insulin independence, but non-technical pancreas graft dysfunction remains difficult to anticipate. Methods: We conducted an exploratory single-centre retrospective biomarker-modelling study to determine whether day-zero recipient serum Fourier-transform infrared (FTIR) spectra are associated with subsequent loss of insulin independence after SPK transplantation. Results: Among 104 screened recipients, 51 met predefined sample-availability, spectral-quality, data-linkage and endpoint-adjudication criteria; 30 maintained pancreas graft function and 21 developed dysfunction. Cases dominated by early technical surgical failure were excluded. Clinical-only, FTIR-only and FTIR–clinical Naïve Bayes models were evaluated using leave-one-out cross-validation with Fast Correlation-Based Filter feature selection. In locked-feature internal validation, the best FTIR-only model used second-derivative spectra with vector normalization and nine selected wavenumbers, achieving AUC 0.997 (95% CI 0.985–1.000) and accuracy 0.961 (95% CI 0.902–1.000). A fixed-feature permutation analysis exceeded label-randomized performance (empirical *p* = 0.001). The secondary Group 1 versus Group 3 analysis suggested discrimination of pancreas dysfunction despite preserved kidney function (AUC 0.992; accuracy 0.930). Conclusions: Given the small cohort, high-dimensional input, non-nested feature selection, selection-bias risk and absence of external validation, serum FTIR should be considered a candidate risk-enrichment platform requiring prospective multicentre validation.

## 1. Introduction

Simultaneous pancreas–kidney (SPK) transplantation is the most established transplant strategy for selected patients with insulin-dependent diabetes and end-stage kidney disease. By restoring renal function and endogenous insulin secretion, SPK transplantation can provide insulin independence, improve glycaemic stability and reduce the clinical burden of diabetes and dialysis. Contemporary registry analyses and international consensus statements show that outcomes after pancreas transplantation have improved substantially over time, particularly after SPK transplantation, where pancreas graft survival remains superior to pancreas transplant alone and pancreas-after-kidney transplantation in most contemporary settings [1,2,3,4].

Despite these improvements, pancreas graft dysfunction remains a clinically important and biologically heterogeneous event. Some early pancreas graft losses are dominated by unequivocal technical or mechanical causes, including vascular thrombosis, anastomotic complications, severe haemorrhagic events, graft pancreatitis requiring surgical intervention or graft pancreatectomy. These events are clinically important, but they may represent direct surgical/technical failure rather than a recipient-side biochemical vulnerability state. Other patients lose or fail to maintain endocrine graft function after initial transplantation through less immediately mechanical trajectories involving ischemia–reperfusion injury, pancreatitis, infection, recurrent autoimmunity, T cell-mediated rejection, antibody-mediated rejection, drug-related metabolic effects or progressive endocrine graft failure [5,6,7,8,9,10,11,12,13,14,15]. For biomarker discovery, this distinction is essential because a day-zero serum test should not be expected to replace surgical adjudication of direct technical graft loss.

Monitoring pancreas graft function is intrinsically difficult. Serum amylase and lipase are widely used but lack specificity for graft injury. Hyperglycaemia may occur late and can be confounded by infection, corticosteroids, nutritional status and insulin resistance. C-peptide and HbA1c provide information about endocrine performance but do not necessarily identify the mechanism, timing or reversibility of injury. Histological assessment remains central when rejection is suspected, and the Banff pancreas allograft schema provides the most widely accepted clinicopathological framework for T cell-mediated rejection, antibody-mediated rejection, mixed rejection, islet pathology and non-rejection lesions [13,14]. However, pancreas graft biopsy is invasive, technically demanding, variably implemented across transplant programs and not suitable as a high-frequency screening test [13,14,16,17,18]. These limitations have stimulated interest in minimally invasive biomarkers capable of detecting graft injury or graft vulnerability earlier than conventional clinical markers [6,7,16,17,18,19].

Serum FTIR spectroscopy offers a complementary biomarker strategy. Unlike single-analyte assays, FTIR captures an integrated vibrational fingerprint of the circulating biochemical milieu. Serum spectra contain overlapping molecular contributions from proteins, lipoproteins, lipids, carbohydrates, phosphates, nucleic-acid-associated moieties and low-molecular-weight metabolites. FTIR-based biofluid diagnostics has been explored across several clinical contexts within established biomarker-development frameworks [20], because it is rapid, reagent-free, relatively inexpensive and potentially compatible with high-throughput analytical workflows [20,21,22,23,24]. However, clinical translation of FTIR-based biomarkers requires rigorous pre-analytical control, transparent reporting, independent validation, inter-instrument harmonization and careful protection against overfitting in high-dimensional spectral datasets [20,21,22,23,24,25,26,27].

In transplantation, our group has recently shown that serum FTIR spectroscopy combined with machine learning can discriminate kidney allograft rejection phenotypes from non-rejection states [28,29]. This provided methodological precedent that allograft injury and immune activation can be associated with detectable systemic biochemical patterns. The present study addresses a distinct clinical problem: pancreas graft dysfunction/loss of insulin independence after SPK transplantation. The kidney component is used here to contextualize SPK transplantation and to construct a secondary phenotype analysis designed to determine whether FTIR can identify pancreas dysfunction even when kidney graft function remains preserved.

The primary aim of this study was to determine whether day-zero recipient serum FTIR spectra can discriminate maintained pancreas graft function from subsequent clinically adjudicated functional pancreas graft dysfunction, defined by loss of insulin independence, after SPK transplantation. Cases whose outcome was dominated by early perioperative or immediate postoperative technical complications were excluded from the primary biomarker-discovery endpoint to avoid training the classifier on direct mechanical/surgical failures. The secondary aim was to compare FTIR-only models with clinical-only and combined FTIR–clinical models. Finally, we explored whether FTIR could discriminate recipients with preserved pancreas and kidney graft function from recipients with pancreas dysfunction despite preserved kidney graft function.

## 2. Materials and Methods

### 2.1. Study Design and Cohort Assembly

This was an exploratory single-centre retrospective biomarker-modelling study of SPK transplant recipients screened for day-zero serum FTIR analysis. The source screened cohort comprised 104 SPK transplant recipients with potential eligibility for FTIR-based biomarker assessment. This number represents the screened clinical/biobank cohort and not the final modelling dataset. Exclusions were applied before any supervised modelling, independently of classifier performance, and according to predefined sample-availability, analytical-quality, data-linkage and endpoint-adjudication requirements. Recipients whose pancreas graft outcome was dominated by early perioperative or immediate postoperative technical complications were removed from the biomarker-discovery endpoint, because these events were adjudicated as direct mechanical/surgical causes of graft failure rather than biologically interpretable functional vulnerability phenotypes.

**Figure 1:**
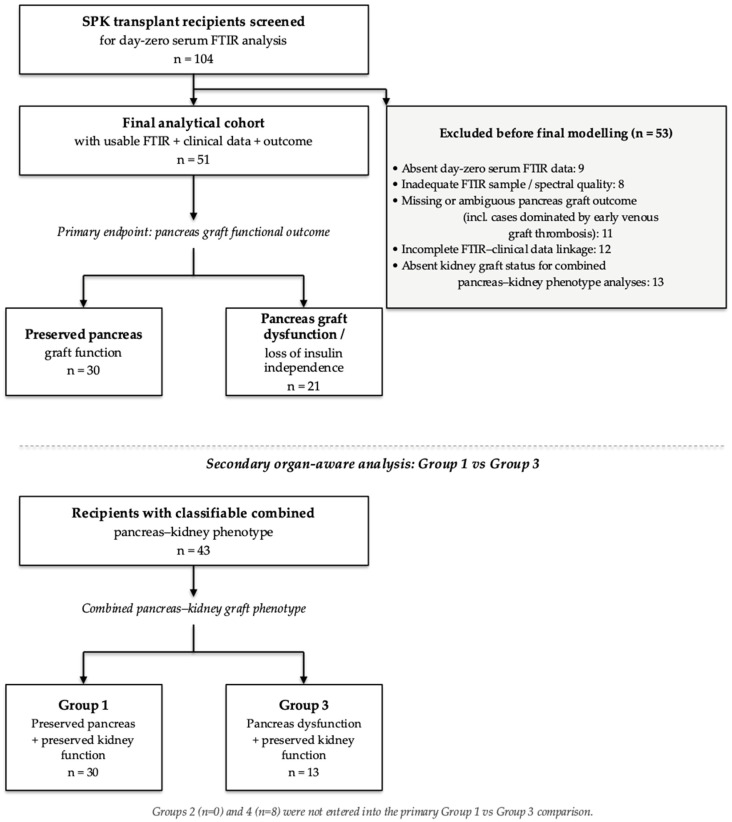
Cohort flowchart and analytical subsets. A total of 104 SPK transplant recipients were screened for potential day-zero serum FTIR-based biomarker analysis. Exclusions were applied before supervised modelling and were based on sample availability, FTIR analytical quality, clinical data linkage and endpoint adjudication. Fifty-three cases were not entered into the final primary modelling dataset because of absence of day-zero serum FTIR data (n = 9), inadequate FTIR sample/spectral quality (n = 8), missing or ambiguous pancreas graft functional outcome or endpoint adjudication dominated by early perioperative/immediate postoperative technical complications (n = 11), incomplete linkage between FTIR and clinical data (n = 12) or absence of kidney graft status for analyses requiring combined pancreas–kidney phenotype classification (n = 13). The final primary analytical cohort included 51 recipients. The secondary Group 1 versus Group 3 analysis included 43 recipients (Figure 1).

After cohort adjudication, the final primary analytical cohort included 51 recipients with usable day-zero FTIR spectra, linked peri-transplant clinical data and classifiable pancreas graft functional outcome. Within this cohort, 30 recipients were classified as having preserved pancreas graft function and 21 as having pancreas graft dysfunction/loss of insulin independence (Figure 1).

For the secondary pancreas–kidney phenotype analysis, 43 recipients were included in the Group 1 versus Group 3 comparison: 30 Group 1 cases and 13 Group 3 cases. Cases with missing kidney graft status were considered ineligible for combined pancreas–kidney phenotype analyses, but kidney status was not part of the biological definition of the primary pancreas functional endpoint.

### 2.2. Eligibility Criteria

The inclusion criteria for the primary analysis were as follows: (1) recipient of SPK transplantation at the study institution; (2) availability of a day-zero recipient serum sample analysed by FTIR spectroscopy; (3) FTIR sample/spectrum meeting analytical quality requirements; (4) availability of linked clinical and peri-transplant data; and (5) availability of a classifiable pancreas graft functional outcome based on post-transplant insulin independence. For the secondary combined pancreas–kidney phenotype analysis, availability of kidney graft status was additionally required.

The exclusion criteria were as follows: (1) absence of day-zero serum FTIR data; (2) inadequate FTIR sample or spectral quality; (3) missing or ambiguous pancreas graft functional outcome; (4) pancreas graft loss dominated by early perioperative or immediate postoperative technical complications, including direct surgical/mechanical graft failure; (5) incomplete linkage between FTIR and clinical data; and (6) absence of kidney graft status for analyses requiring combined pancreas–kidney phenotype classification. Exclusions were applied before model training and were not informed by cross-validation results or classifier performance.

### 2.3. Outcome Definition

The primary endpoint was clinically adjudicated pancreas graft functional status after exclusion of cases dominated by early technical perioperative or immediate postoperative graft failure. Preserved pancreas graft function was defined as maintained insulin independence after transplantation, without return to exogenous insulin therapy during follow-up. The median follow-up of the analytical cohort was 4.6 years [IQR 3.3–6.4]. Median follow-up was 5.0 years [IQR 4.2–6.9] in recipients with preserved pancreas graft function and 3.5 years [IQR 2.2–6.2] in recipients with pancreas graft dysfunction/loss of insulin independence. Follow-up duration was compared descriptively between groups because shorter follow-up in the dysfunctional group may occur when the event is defined by return to exogenous insulin therapy; the difference did not reach statistical significance (Mann–Whitney U, *p* = 0.076).

Pancreas graft dysfunction was defined as loss of pancreas graft endocrine function, operationalized as return to exogenous insulin therapy at any point during follow-up after previous graft function, or failure to maintain insulin independence, according to the final clinically adjudicated pancreas graft status.

This endpoint was interpreted as clinically relevant functional graft dysfunction/loss of insulin independence rather than a transient perioperative insulin-requirement phenotype or a direct technical graft-failure phenotype. Short protocol-driven insulin use in the immediate perioperative period was not considered sufficient to define dysfunction. Conversely, recipients whose graft outcome was primarily explained by early perioperative or immediate postoperative technical complications were not used to train the primary biomarker model, because these events were considered direct mechanical/surgical failures rather than recipient-side biochemical vulnerability states. The endpoint therefore identifies recipients who lost or failed to maintain endocrine graft function over follow-up, while acknowledging that the underlying non-technical mechanisms may include pancreatitis, ischemia–reperfusion injury, infection, immune-mediated injury, recurrent autoimmunity or progressive endocrine graft failure. More granular definitions of beta-cell graft function have been proposed, including the Igls criteria, which classify beta-cell replacement outcomes according to HbA1c, severe hypoglycaemia, insulin requirement and C-peptide production [19,30]. In the present study, the clinically adjudicated outcome was intentionally pragmatic and based on the most clinically meaningful functional event: loss of insulin independence.

### 2.4. Combined Pancreas–Kidney Phenotype Groups

For the secondary analysis, recipients were classified according to combined pancreas and kidney graft status. This grouping was used to test whether the FTIR signal could discriminate pancreas dysfunction in the presence of preserved kidney function (Table 1).

The principal secondary analysis compared Group 1 versus Group 3. This comparison was deliberately selected because Group 3 represents pancreas dysfunction despite preserved kidney function, thereby reducing the possibility that the FTIR signal merely reflects renal dysfunction or nonspecific dual graft failure.

No recipient in the final analytical cohort fulfilled the Group 2 phenotype, defined as preserved pancreas graft function with kidney graft dysfunction. This distribution may reflect the clinical course and selection structure of the analysed cohort, but it prevents assessment of whether FTIR can distinguish isolated renal dysfunction from preserved dual graft function in this dataset. Therefore, the secondary phenotype analysis was restricted to Group 1 versus Group 3 and should not be interpreted as a complete organ-specific decomposition of pancreas and kidney dysfunction.

### 2.5. Clinical Variables

The clinical dataset included recipient, donor, immunological and perioperative variables. Candidate predictors comprised recipient age, sex, anthropometrics, previous transplantation, dialysis modality and duration, diabetes duration and treatment, ethnicity, diabetic microvascular and macrovascular complications, cardiovascular disease, vascular disease, sensitization variables, HLA mismatch variables, pre-transplant donor-specific antibody status, HbA1c, donor age, donor sex, donor anthropometrics, cause of death, donor ventilation, donor vasoactive support, donor inflammatory and pancreatic enzyme markers, operative duration, cold ischemia time, warm ischemia time, intraoperative blood transfusion and ASA class. The final clinical modelling file contained 53 candidate clinical features.

### 2.6. FTIR Acquisition and Preprocessing

Recipient serum was collected at day zero within the peri-transplant pathway and processed according to the centre’s standard biobanking workflow before FTIR analysis. Spectral data were collected using an FTIR spectrometer (Vertex 70, Bruker, Mannheim, Germany) equipped with a high-throughput screening accessory (HTS-XT, Bruker, Mannheim, Germany) in transmission mode, spanning 400–4000 cm^−1^, with 32 co-added scans at 2 cm^−1^ resolution. To minimize plate-related bias, sample placement was randomized and/or alternated across the plate to reduce spatial/edge effects, and sample identifiers were masked during preprocessing and downstream analyses. All spectra were acquired on the same instrument using identical parameters and environmental conditions whenever feasible.

Spectral data were exported as high-dimensional absorbance variables spanning the mid-infrared region, approximately 4000–600 cm^−1^ in the final analytical files [21,23,24]. The final FTIR spectral dataset initially comprised 3733 wavenumber variables per recipient. After restriction to the analytical spectral region used for modelling, the FTIR-only input matrix included 51 recipients and 1727 candidate spectral variables before feature selection. Several preprocessing strategies were evaluated, including raw spectra, baseline correction, rubber-band baseline correction, vector normalization, first-derivative transformation and second-derivative transformation. The best-performing FTIR-only model was obtained using vector-normalized second-derivative spectra. Following FCBF feature selection, the FTIR-only feature space was reduced from 1727 candidate spectral variables to nine selected wavenumbers, which were subsequently used as classifier inputs.

### 2.7. Spectral Quality-Control Assessment

A spectral quality-control layer was applied before interpretation of supervised models to assess whether downstream separation could plausibly be driven by technical differences between groups. Four complementary metrics were computed for each spectrum: (1) baseline drift index, summarized as a percentage of the absolute spectral area; (2) cosine similarity between each spectrum and the cohort median spectrum; (3) signal-to-noise ratio in the Amide I region; and (4) spike-like artefact burden in the fingerprint region, quantified using first-difference outlier counts with a median absolute deviation threshold of |z| > 6.

Quality-control metrics were summarized overall and by the pancreas outcome group. Between-group comparisons were exploratory and were used to evaluate potential technical imbalance, not to select or remove samples after outcome labels were known. Unsupervised analyses, including multidimensional scaling (MDS) based on cosine distance and hierarchical cluster analysis with cosine-distance heatmap, were used as exploratory diagnostics of latent spectral structure and potential artefact-driven separation. These unsupervised visualizations were not used for classifier training and should not be interpreted as model validation.

For t-SNE visualizations, background colour shading was added only as a qualitative two-dimensional kernel-density/smoothed occupancy overlay to facilitate visual appreciation of local point concentration by class. This shading was not derived from the classifier, does not represent class probability or a decision boundary, and should not be interpreted quantitatively.

### 2.8. Feature Selection and Machine-Learning Classification

Feature selection was performed using Fast Correlation-Based Filter (FCBF), a correlation-based filter method designed to remove irrelevant and redundant predictors in high-dimensional datasets [27]. FCBF was applied separately to clinical-only, FTIR-only and FTIR–clinical feature spaces.

Naïve Bayes classifiers were trained and evaluated using leave-one-out cross-validation (LOOCV). FCBF was used as a filter-based feature-selection method to derive compact clinical, FTIR-only and FTIR–clinical candidate feature sets. After the second-round validation audit, the final FTIR-only performance estimates were reported using a locked FCBF-selected spectral feature set. Within each LOOCV fold, fold-dependent operations required for the classifier, including quantile discretization and Naïve Bayes parameter estimation, were fitted using only the training observations and the held-out observation was then classified. FCBF feature selection was applied as a global filter step to the analytical cohort prior to the LOOCV loop, and the locked feature set was held fixed across folds. Therefore, the reported LOOCV performance evaluates classifier fitting and prediction using a pre-selected locked feature set, but it does not fully estimate the performance of a completely nested feature-selection-plus-classification pipeline. We acknowledge that this design may produce optimistic estimates of generalization performance, particularly in a small high-dimensional cohort. Independent external validation is therefore required (see Section 4.2). Spectrum-level preprocessing operations, including derivative transformation and vector normalization, were deterministic per-spectrum transformations and did not use outcome labels. Because this was a small high-dimensional exploratory study, all performance estimates were interpreted as internal validation results rather than definitive clinical validation. Reporting was organized according to STROBE and TRIPOD + AI principles [25,26].

### 2.9. Statistical Analysis

Continuous variables are summarized as median [interquartile range] or mean ± standard deviation according to distribution. Categorical variables are summarized as n/N (%), excluding missing values from denominators. Exploratory unadjusted comparisons between preserved pancreas function and pancreas graft dysfunction groups were performed using Mann–Whitney U tests for continuous variables and Fisher exact or chi-square tests for categorical variables, as appropriate. PRA CDC maximum was analysed as a continuous/rank-based variable using the Mann–Whitney U test, according to the pre-specified approach for continuous variables. Because its distribution was highly zero-inflated, the resulting *p*-value was interpreted descriptively and cautiously. No additional inferential claim was made from this variable. Where data were available, screened recipients who entered the final analytical cohort were descriptively compared with excluded recipients to assess potential gross selection imbalance; these comparisons were considered selection-bias diagnostics and were not used for feature selection or model inference. All *p*-values are descriptive only and were not adjusted for multiple comparisons. Model metrics included AUC, classification accuracy, precision, recall and specificity. For the final FTIR-only model, 95% confidence intervals for AUC and accuracy were estimated by stratified non-parametric bootstrap resampling of the LOOCV prediction set with 10,000 bootstrap replicates. To evaluate whether the observed discrimination exceeded chance-level performance in the high-dimensional setting, a permutation/y-randomization analysis was performed with 1000 random permutations of the outcome labels, retraining the Naïve Bayes classifier on the locked feature set within each permutation. Because FCBF feature selection was applied to the analytical cohort before the LOOCV loop and was not re-executed within each permutation, the resulting empirical *p*-value should be interpreted as a conditional test of classifier performance given the locked FCBF-selected feature set, rather than as a test of the entire feature-selection-plus-classification pipeline. This fixed-feature permutation design does not eliminate the possibility of feature-selection optimism. The empirical permutation *p*-value was calculated as (1 + number of permuted AUC values greater than or equal to the observed AUC)/(1 + number of permutations).

No external validation cohort was available.

### 2.10. Ethics

The study was conducted in accordance with the Declaration of Helsinki. Clinical data were retrieved retrospectively from the institutional clinical database and linked to stored serum samples from the institutional biobank. Participants included in the biobank-linked cohort had provided prospective informed consent allowing future research use of stored biological samples and associated clinical data. The retrospective analysis was approved by the ULS Ethics Committee (01/2021/CEFCM).

## 3. Results

### 3.1. Cohort Flow

A total of 104 SPK transplant recipients were screened for potential day-zero serum FTIR-based biomarker analysis between 2011 and 2022. Fifty-three cases were not entered into the final primary modelling dataset because they failed predefined analytical, linkage or endpoint-adjudication requirements. Exclusions were applied before supervised modelling and were not informed by model performance. The exclusion audit comprised absence of day-zero serum FTIR data (n = 9), inadequate FTIR sample/spectral quality (n = 8), missing or ambiguous pancreas graft functional outcome or endpoint adjudication dominated by early perioperative/immediate postoperative technical complications (n = 11), incomplete linkage between FTIR and clinical data (n = 12), and absence of kidney graft status for combined pancreas–kidney phenotype analyses (n = 13). The final analytical cohort included 51 recipients with usable FTIR spectra, linked clinical data and classifiable pancreas graft functional outcome. Among these, 30 recipients had preserved pancreas graft function and 21 had pancreas graft dysfunction/loss of insulin independence. The secondary Group 1 versus Group 3 analysis included 43 recipients, comprising 30 Group 1 cases and 13 Group 3 cases. Because 53 of 104 screened recipients were not included in the final primary modelling dataset, the analytical cohort may not fully represent the overall SPK population screened for this study. These exclusions were applied before supervised modelling and were not informed by classifier performance, but they remain a potential source of selection bias and limit generalizability. In addition, the final cohort retained a moderate class imbalance, with 30 recipients with preserved pancreas graft function and 21 recipients with pancreas graft dysfunction/loss of insulin independence. We therefore avoided post hoc expansion of the preserved-function group alone, as this would have increased outcome imbalance and could have further biased internal performance estimates. The final analytical set should consequently be interpreted as a constrained exploratory modelling cohort rather than as a fully representative SPK transplant population.

### 3.2. Baseline Characteristics

Baseline and perioperative characteristics of the final 51-recipient analytical cohort are summarized in Table 2. Groups were broadly comparable across most recipient and donor characteristics. In exploratory unadjusted comparisons, pancreas graft dysfunction was associated with a higher frequency of femoral-iliac calcification/stenosis on angio-CT and a lower frequency of donor noradrenaline use. PRA CDC maximum differed between groups in the exploratory rank-based comparison; however, the variable was highly zero-inflated, with medians of 0 in both groups. Therefore, this *p*-value should be interpreted as descriptive evidence of distributional imbalance rather than as evidence of a robust difference in central tendency. Because substantial pre-modelling exclusions were required, baseline comparisons should be interpreted as descriptive characterization of the final analytical cohort rather than proof of absence of selection bias.

### 3.3. Spectral Assessment

**Figure 2:**
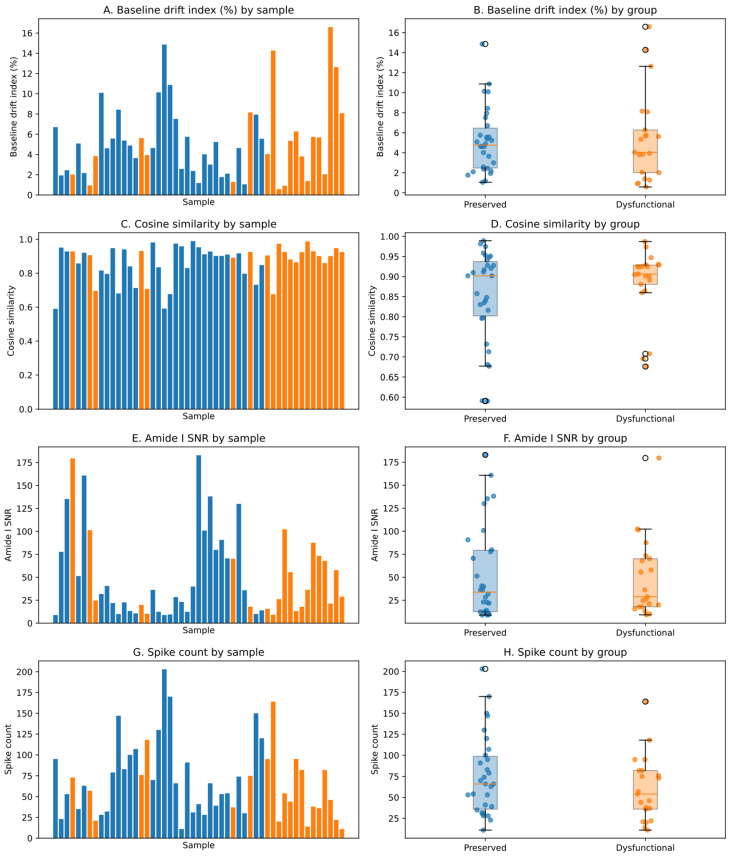
Spectral quality-control metrics by pancreas graft outcome group. Baseline drift index, cosine similarity to the cohort median spectrum, Amide I signal-to-noise ratio and spike-like artefact burden were summarized for preserved and dysfunctional groups. No QC metric showed a significant group imbalance.

Spectral QC metrics were broadly comparable between preserved and dysfunctional pancreas graft outcome groups (Table 3 and Figure 2). Baseline drift, cosine similarity to the cohort median spectrum, Amide I signal-to-noise ratio and spike-like artefact burden showed no significant group imbalance. This supports the interpretation that downstream class separation was unlikely to be primarily driven by systematic spectral quality differences between groups.

### 3.4. Unsupervised Spectral Structure

**Figure 3:**
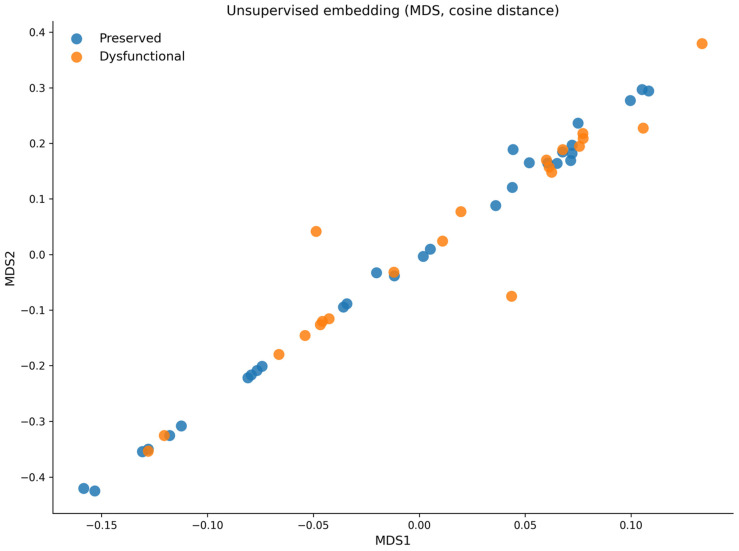
Unsupervised MDS embedding using cosine distance. MDS was computed from the final spectral feature space using cosine distance. The plot was used as an exploratory diagnostic for latent structure and potential outliers, not as supervised evidence of model performance.

**Figure 4:**
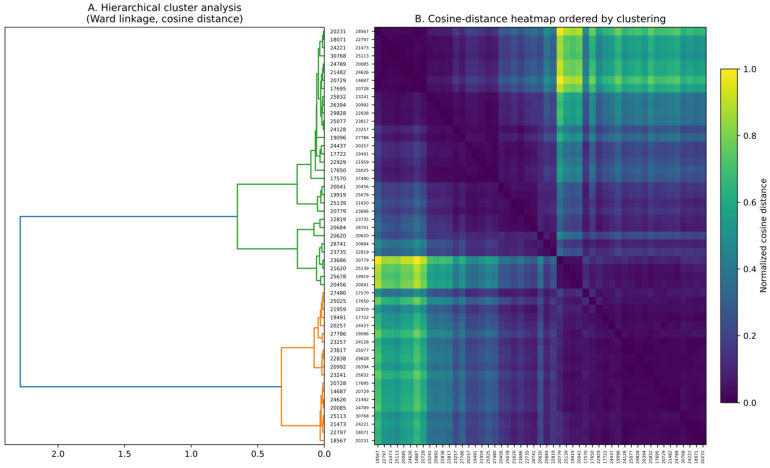
Hierarchical cluster analysis and cosine-distance heatmap. Hierarchical cluster analysis was performed using cosine distance and an exploratory linkage approach. The heatmap is ordered by clustering and highlights block structure with lower within-cluster and higher between-cluster distances.

Unsupervised analyses were performed to evaluate intrinsic spectral structure without using outcome labels for supervised learning. MDS using cosine distance showed partial overlap between preserved and dysfunctional spectra, without dominant group-driven clustering suggestive of acquisition drift (Figure 3). Hierarchical cluster analysis using cosine distance showed two main spectral branches, and the corresponding cosine-distance heatmap showed block structure with lower within-cluster and higher between-cluster distances (Figure 4). These findings support the presence of non-random multivariate spectral organization while reinforcing that supervised classification was required to integrate subtle distributed signals.

### 3.5. FTIR-Only Modelling

**Figure 5:**
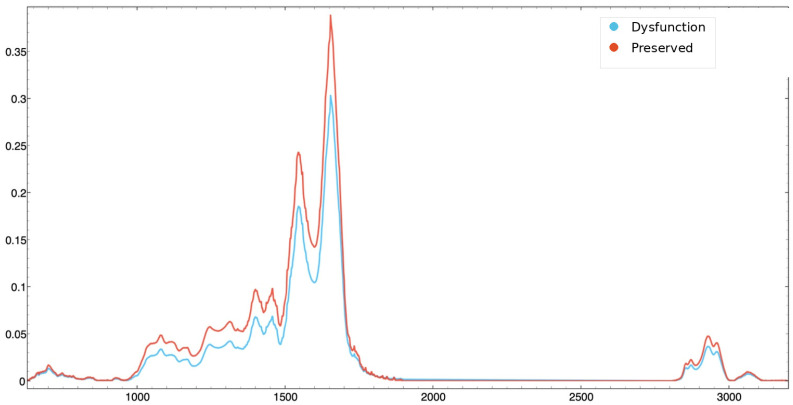
Representative FTIR spectral profiles after baseline correction and normalization. Representative serum FTIR spectral profiles after preprocessing. The figure illustrates the spectral regions used for downstream modelling. Spectral visualization is descriptive and was not used as a stand-alone classifier.

FTIR-only performance varied according to spectral preprocessing (Figure 5). Raw FTIR with FCBF yielded modest discrimination, whereas derivative-based and normalized spectral representations improved performance. The best FTIR-only model used second-derivative spectra with vector normalization and FCBF-selected wavenumbers.

**Figure 6:**
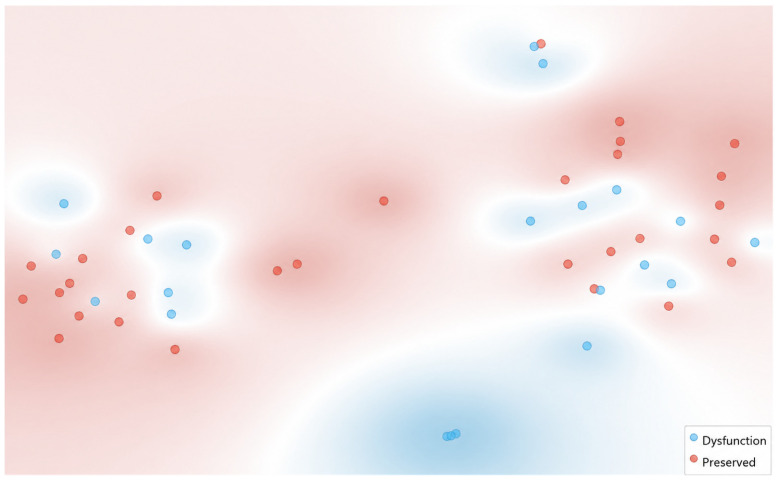
t-SNE projection of the FTIR-only feature space corresponding to second-derivative spectra with vector normalization and FCBF-selected wavenumbers. Each point represents one recipient and is coloured according to pancreas graft functional outcome. Background shading represents a qualitative smoothed density overlay of the projected points by class and is shown only for visual orientation; it does not represent a classifier-derived probability surface, decision boundary or quantitative estimate.

In the final locked FTIR-only validation analysis, from the initial 1727-variable spectral matrix, FCBF selected nine wavenumbers for the final locked FTIR-only model, ≈1301, ≈3033, ≈1224, ≈2960, ≈3113, ≈1098, ≈879, ≈843 and ≈1119 cm^−1^. Using fold-wise classifier fitting in LOOCV, this FTIR-only model achieved AUC 0.997 (95% CI 0.985–1.000), classification accuracy 0.961 (95% CI 0.902–1.000), precision 0.964, recall 0.961 and specificity 0.973. It correctly classified 21/21 pancreas dysfunction cases and 28/30 preserved pancreas cases (Figure 6).

Permutation testing with 1000 label-randomized repetitions showed that, conditional on the locked FCBF-selected feature set, the observed FTIR-only AUC was higher than the AUC distribution obtained after label randomization (empirical *p* = 0.001).

### 3.6. Clinical-Only and Combined FTIR–Clinical Modelling

The clinical-only model using all 53 available clinical features had limited discriminative ability, with AUC 0.516, classification accuracy 0.569, precision 0.510, recall 0.569 and specificity 0.427. After FCBF feature selection, the selected clinical model improved to AUC 0.860 and classification accuracy 0.804. The corresponding confusion matrix showed correct classification of 14/21 pancreas dysfunction cases and 27/30 preserved pancreas cases. The six FCBF-selected clinical variables were PRA CDC maximum (%), donor noradrenaline use, ischemic stroke, femoral-iliac calcification/stenosis, dyslipidaemia and inflammatory bowel disease. The marked improvement after FCBF should be interpreted cautiously. The all-variable clinical model included 53 candidate clinical features in only 51 recipients, creating an unfavourable predictor-to-sample-size ratio with potential redundancy, sparsity and noise. FCBF reduced this feature space to a small subset of variables, which may improve apparent internal discrimination, but in this small cohort it may also introduce feature-selection optimism. Therefore, the selected clinical-only model is presented as an exploratory comparator rather than as a validated clinical prediction model.

**Figure 7:**
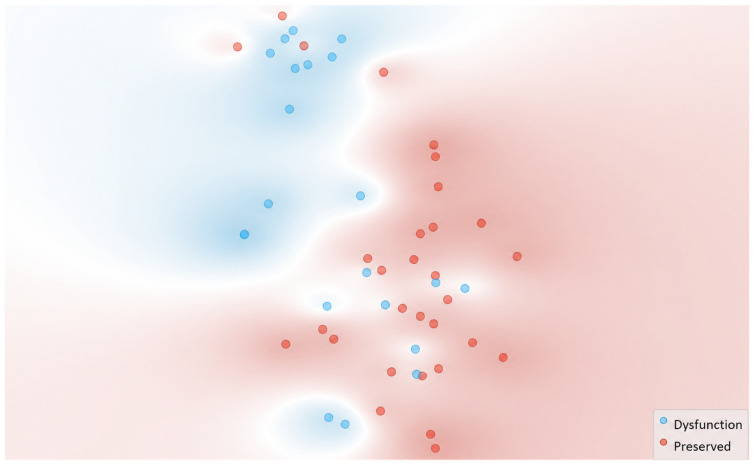
t-SNE visualization of the FTIR–clinical feature space. t-SNE projection of the combined FTIR–clinical feature space selected by FCBF. Each point represents one recipient and is coloured according to pancreas graft functional outcome. Background shading represents a qualitative smoothed density overlay of the projected points by class and is shown only for visual orientation; it does not represent a classifier-derived probability surface, decision boundary or quantitative estimate. This visualization is exploratory and should not be interpreted as model validation.

The combined FTIR–clinical model selected both spectral and clinical features, including PRA CDC maximum, ischemic stroke and dyslipidaemia, together with wavenumbers around 843.778, 1098.358, 1223.720, 1300.865, 3012.530 and 3112.819 cm^−1^. The combined model achieved AUC 0.990, classification accuracy 0.961, precision 0.963, recall 0.961 and specificity 0.944. Performance of the combined model was excellent but slightly below the FTIR-only model, suggesting that the dominant discriminative information was already present in the serum FTIR spectral profile and that adding clinical variables did not provide incremental discriminative value in this cohort (Figure 7).

### 3.7. Secondary Analysis: Group 1 Versus Group 3

The secondary analysis compared recipients with preserved pancreas and kidney graft function (Group 1) against recipients with pancreas graft dysfunction but preserved kidney graft function (Group 3). This analysis included 43 recipients: 30 in Group 1 and 13 in Group 3.

**Figure 8:**
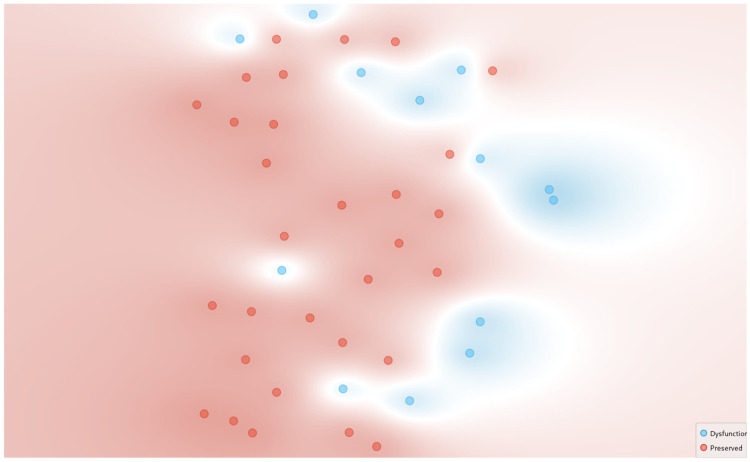
t-SNE visualization of the Group 1 versus Group 3 comparison. t-SNE projection for the secondary analysis comparing Group 1, defined as preserved pancreas and kidney function, with Group 3, defined as pancreas graft dysfunction with preserved kidney function. Each point represents one recipient. Background shading represents a qualitative smoothed density overlay of the projected points by group and is shown only for visual orientation; it does not represent a classifier-derived probability surface, decision boundary or quantitative estimate.

The FTIR model achieved AUC 0.992, classification accuracy 0.930, precision 0.930, recall 0.930 and specificity 0.883. The confusion matrix showed correct classification of 11/13 Group 3 cases and 29/30 Group 1 cases. This result supports the hypothesis that the FTIR signature is associated with pancreas dysfunction even when kidney graft function remains preserved (Figure 8).

### 3.8. Comparative Model Performance

The performance of the principal models, together with the corresponding confusion-matrix readouts, is summarized in Table 4, and the FCBF-selected variables for the best-performing spectral and combined models are listed in Table 5.

## 4. Discussion

This exploratory single-centre study suggests that day-zero recipient serum FTIR spectroscopy contains a strong multivariate biochemical signature associated with subsequent clinically adjudicated, non-technical functional pancreas graft dysfunction/loss of insulin independence after SPK transplantation. The central finding is that, in internal locked-feature validation, the best FTIR-only model showed very high discriminative performance and outperformed both clinical variables alone and the combined FTIR–clinical model. Because feature selection was performed before LOOCV and no external validation cohort was available, this performance should be interpreted as exploratory internal discrimination rather than as definitive evidence of clinical generalizability. This supports the hypothesis that serum FTIR captures integrated biochemical information not fully represented by conventional recipient, donor, immunological and perioperative variables. Importantly, cases dominated by early perioperative or immediate postoperative technical complications were not used to define the primary biomarker-discovery endpoint, preserving the interpretation of the model as a functional vulnerability signal rather than a detector of direct surgical/mechanical graft failure.

The endpoint used in this study was intentionally functional and clinically pragmatic. Insulin independence is one of the principal clinical goals of pancreas transplantation and return to exogenous insulin therapy is frequently used in registry and clinical definitions of pancreas graft functional loss. In contrast to a transient perioperative insulin-requirement phenotype, the dysfunctional group in this study comprised recipients who lost or failed to maintain pancreas endocrine graft function during follow-up, reflected by return to exogenous insulin therapy according to the final clinically adjudicated graft status. In contrast to early technical graft failure, this endpoint was intended to capture functional loss potentially related to recipient-side vulnerability, graft injury biology or progressive endocrine failure. This distinction is essential for translational interpretation: the model should be viewed as a risk-stratification signal for clinically relevant loss of pancreas graft function, not as a stand-alone rejection assay, a mechanism-specific diagnostic test or a predictor of unavoidable technical complications.

The spectral QC analysis is important because it supports the analytical credibility of the results. Baseline drift, cosine similarity to the cohort median spectrum, Amide I SNR and spike-like artefact burden did not differ significantly between preserved and dysfunctional groups. This reduces the likelihood that the observed model performance was driven by systematic technical artefacts or sample-quality imbalance. In serum vibrational spectroscopy, this step is critical because subtle disease-associated differences can be confounded by baseline distortion, film-thickness variation, drying effects, scattering or acquisition artefacts. The additional unsupervised MDS and hierarchical clustering analyses showed non-random spectral structure but no dominant artefact-like separation, supporting the interpretation that the supervised signal is distributed and multivariate rather than a gross batch effect.

The selected wavenumbers should not be interpreted as individually identified biochemical biomarkers. Serum FTIR spectra represent overlapping contributions from proteins, lipoproteins, lipids, carbohydrates, phosphates, nucleic-acid-associated moieties and low-molecular-weight metabolites. In addition, second-derivative preprocessing can redistribute spectral information and complicate direct band attribution. Because the selected wavenumbers were chosen by a data-driven filter rather than by a predefined molecular hypothesis, they are best regarded as discriminative spectral surrogates within a multicomponent serum fingerprint, not as molecule-specific markers.

This systems-level interpretation is biologically plausible in SPK transplantation. The pancreas allograft is uniquely vulnerable to ischemia–reperfusion injury, endothelial activation, microvascular disturbance, thrombosis, oedema, pancreatitis and immune-mediated injury. These processes do not act in isolation; rather, they converge through thrombo-inflammatory, metabolic and vascular pathways. Day-zero recipient serum FTIR may capture a pre-existing biochemical state shaped by long-standing diabetes, uraemia, glycation, lipoprotein remodelling, chronic vascular disease, inflammation, complement/coagulation biology and protein conformational or hydration-related changes. Such a state may not directly cause graft dysfunction, but it may modulate susceptibility to perioperative injury and transient endocrine instability after reperfusion.

The Group 1 versus Group 3 analysis strengthens the pancreas-focused interpretation. Group 3 represents pancreas dysfunction despite preserved kidney graft function. Therefore, discrimination between Group 1 and Group 3 is less likely to be explained solely by renal dysfunction or generalized dual graft failure. This does not prove pancreas specificity at the molecular level, but it supports the presence of a pancreas-associated serum biochemical signal. In other words, the FTIR profile appears to reflect a risk phenotype related to pancreas graft vulnerability, not simply a marker of global graft failure.

The observation that FTIR–clinical modelling did not materially outperform FTIR-only modelling is also relevant. One interpretation is that the spectral fingerprint already compresses several dimensions of recipient biological risk that are only partially reflected in standard clinical variables. Another possibility is that the sample size was too small for clinical variables to provide stable incremental information when added to high-dimensional spectral features. These explanations are not mutually exclusive. For clinical translation, however, the finding is attractive: if externally validated, a serum-only workflow could offer a low-burden, rapid and potentially scalable front-line risk-stratification tool.

The translational role of this candidate biomarker should be defined conservatively. FTIR should not replace biopsy, DSA assessment, imaging, standard biochemical monitoring or emerging molecular tests such as donor-derived cell-free DNA. A direct head-to-head comparison with donor-derived cell-free DNA was not possible in the present retrospective cohort because dd-cfDNA was not measured in parallel with day-zero FTIR profiling. Future prospective validation studies should include paired FTIR and dd-cfDNA sampling to determine whether FTIR provides complementary risk-enrichment information, incremental predictive value or earlier systemic biochemical stratification beyond established molecular injury biomarkers [16]. Its most credible role is as an early triage layer. A low-risk day-zero spectral profile could support standard monitoring, whereas a high-risk profile could justify intensified glucose surveillance, earlier biochemical reassessment, earlier Doppler or cross-sectional imaging when dysfunction emerges and a lower threshold for DSA testing, donor-derived cell-free DNA or graft biopsy if corroborating abnormalities occur. This positions FTIR as a recipient-side surveillance enrichment tool rather than a definitive diagnostic test or organ acceptance tool.

The exclusion of 53 of the 104 screened cases deserves specific discussion. This is one of the main limitations of the study. The exclusions were distributed across absent day-zero FTIR data, inadequate FTIR sample/spectral quality, missing or ambiguous pancreas functional outcome or endpoint adjudication dominated by early perioperative/immediate postoperative technical complications, incomplete FTIR–clinical linkage and absence of kidney graft status for combined phenotype analysis. This explicit reporting is more transparent than a single broad exclusion category, but it does not remove the possibility of selection bias. At the same time, the final sample remains enriched for cases with complete, high-quality, linkable data and a biologically interpretable functional endpoint. Prospective studies should minimize such exclusions through standardized sample acquisition, fixed quality-control thresholds, predefined technical-failure adjudication rules and robust data-linkage procedures. These findings align with previous FTIR studies in kidney transplantation, where serum FTIR combined with machine learning discriminated kidney allograft rejection phenotypes [28,29]. The present study extends that methodological concept to pancreas graft dysfunction after SPK transplantation. The kidney experience should be framed as methodological precedent, not as the biological basis of the present pancreas-focused result. Future work should test whether pancreas-specific endpoints, including biopsy-proven rejection, thrombosis, pancreatitis, delayed endocrine function [31,32] and persistent insulin dependence, map to distinct or overlapping serum FTIR signatures.

### 4.1. Clinical Implications and Translational Pathway

If externally validated, day-zero serum FTIR spectroscopy could become a low-cost, rapid and minimally invasive tool for early pancreas graft risk stratification after SPK transplantation. The most realistic clinical role would be complementary. FTIR could identify recipients with a high-risk serum biochemical signature who warrant intensified follow-up during the perioperative and early post-transplant period.

The translational pathway should proceed in stages. First, the present signal should be reproduced in an independent consecutive cohort using a locked preprocessing, feature-selection and classification pipeline. Second, the model should be recalibrated and tested prospectively with explicit endpoint adjudication. Third, its incremental value should be assessed over standard clinical variables, donor-risk indices, ischemia times, DSA status, early pancreatic enzymes, glycaemic trajectories, imaging and biopsy when available. Fourth, the model should be evaluated as part of a clinical pathway: whether it improves time to diagnostic escalation, enriches the yield of complementary tests, reduces unnecessary investigations in low-risk recipients or identifies high-risk recipients before irreversible graft injury develops.

A clinically useful FTIR model does not need to be a stand-alone diagnostic assay. Its value may lie in risk enrichment. In this framework, FTIR would help move pancreas graft monitoring from a purely reactive model toward a more anticipatory, risk-adapted strategy. This is particularly relevant in SPK transplantation, where early graft injury may evolve rapidly, and where the clinical consequences of delayed recognition include graft thrombosis, pancreatitis, rejection, graft pancreatectomy or prolonged morbidity.

### 4.2. Limitations

This study has several limitations. First, the cohort is small and retrospective. Second, the study is single-centre and therefore reflects local donor selection, surgical practice, anticoagulation protocols, immunosuppression, monitoring strategies and biopsy thresholds. Third, 53 of 104 screened cases were excluded before final modelling, which may limit generalizability and introduce selection bias, even though exclusions were applied before model training and were based on predefined analytical, linkage and endpoint-adjudication requirements. Fourth, exclusion of early technical perioperative or immediate postoperative graft failures strengthens biological interpretability but narrows the target population: the model should not be interpreted as predicting direct surgical/mechanical graft loss. Fifth, there is no external validation cohort. Sixth, although the final locked FTIR-only model was supported by bootstrap confidence intervals and permutation testing, LOOCV in high-dimensional spectral datasets can still overestimate performance in small samples and the FCBF-selected feature set requires external validation in an independent cohort. Seventh, FTIR band assignments are tentative and cannot be interpreted as direct molecular identification. Eighth, the endpoint was functional and clinically pragmatic, but not equivalent to biopsy-adjudicated rejection, thrombosis, pancreatitis or definitive graft failure. Finally, the model has not yet been calibrated, assessed by decision-curve analysis or tested prospectively in a clinical pathway.

In addition, the permutation analysis was performed using the locked FCBF-selected feature set. Therefore, the empirical *p*-value evaluates whether classifier performance exceeded label-randomized performance conditional on those selected features, but it does not validate the complete feature-selection-plus-classification pipeline. This distinction is particularly important given the small sample size and high-dimensional spectral input. These limitations do not negate the signal observed, but they define the required next phase. The present study should be understood as exploratory biomarker discovery and internal signal detection. Clinical implementation would require external validation, inter-instrument harmonization, predefined spectral QC criteria, locked modelling, transparent reporting and prospective demonstration of incremental clinical utility.

## 5. Conclusions

Day-zero recipient serum FTIR spectroscopy identified a compact biochemical signature strongly associated with subsequent clinically adjudicated functional pancreas graft dysfunction after SPK transplantation, defined by loss of insulin independence and return to exogenous insulin therapy during follow-up, after excluding cases dominated by early technical perioperative or immediate postoperative graft failure. The final locked FTIR-only model achieved very high internal discrimination with bootstrap-derived uncertainty estimates, and a fixed-feature permutation analysis supported performance beyond label-randomized expectation. However, because feature selection was not nested within the validation loop and no external validation cohort was available, these results should be interpreted as exploratory internal signal detection. The best FTIR-only model outperformed both clinical variables alone and the combined FTIR–clinical model, indicating that the discriminative signal was concentrated in the spectral profile. The ability to discriminate Group 1 from Group 3 further suggests that the signal is associated with pancreas dysfunction even when kidney graft function is preserved.

The findings support a biologically plausible and clinically relevant concept: recipient serum at day zero contains a multicomponent biochemical fingerprint that may encode vulnerability to non-technical functional pancreas graft dysfunction. This fingerprint should be interpreted as a composite discriminative serum signal rather than as evidence of specific molecule-level biomarkers. FTIR should therefore be positioned as an integrative risk-enrichment platform, not as a stand-alone diagnostic test or a substitute for surgical, radiological, immunological or histological adjudication.

The translational potential is considerable but requires rigorous validation. If reproduced in independent prospective cohorts, day-zero FTIR profiling could become a low-burden, rapid and scalable component of risk-adapted surveillance after SPK transplantation. Its most appropriate role would be to identify recipients requiring intensified monitoring and earlier complementary testing, thereby supporting timely intervention while avoiding overuse of invasive or costly diagnostics in lower-risk recipients. External validation, calibration, inter-laboratory harmonization and prospective clinical utility studies are essential before deployment.

## Figures and Tables

**Table 1:** Combined pancreas–kidney graft phenotype groups.

Group	Pancreas Graft Status	Kidney Graft Status	Interpretation	n
Group 1	Normal/preserved	Normal/preserved	Dual graft preservation	30
Group 2	Normal/preserved	Abnormal/dysfunctional	Renal dysfunction without pancreatic dysfunction	0
Group 3	Abnormal/dysfunctional	Normal/preserved	Isolated pancreatic dysfunction with preserved renal function	13
Group 4	Abnormal/dysfunctional	Abnormal/dysfunctional	Combined pancreatic and renal dysfunction	8
Total	-	-	Final analytical cohort	51

**Table 2:** Baseline recipient, donor and perioperative characteristics by pancreas graft outcome.

Characteristic	Preserved Pancreas Function (n = 30)	Pancreas Graft Dysfunction (n = 21)	*p* Value
Recipient age, years	34 [31–41]	41 [35–45]	0.206
Male recipient sex	19/30 (63.3%)	13/21 (61.9%)	1.000
Recipient BMI, kg/m^2^	22.3 [20.8–24.6]	23.3 [22.0–24.6]	0.339
Previous transplant	2/30 (6.7%)	0/21 (0.0%)	0.506
Haemodialysis	17/30 (56.7%)	14/21 (66.7%)	0.746
Peritoneal dialysis	10/30 (33.3%)	5/21 (23.8%)	0.746
Pre-emptive transplant	3/30 (10.0%)	2/21 (9.5%)	0.746
Dialysis time, days	629 [371–1077]	488 [358–967]	0.373
Diabetes duration, days	9076 [7858–11,078]	9651 [8571–11,931]	0.183
HbA1c pre-transplant, %	8.5 [7.6–9.8]	8.1 [7.7–10.2]	0.840
DSA pre-transplant positive	9/30 (30.0%)	4/17 (23.5%)	0.743
PRA CDC maximum, %	0 [0–9]	0 [0–0]	0.002
vPRA pre-transplant, %	16.4 [0.6–62.1]	6.3 [0.0–76.9]	0.938
Femoral-iliac calcification/stenosis	12/30 (40.0%)	15/20 (75.0%)	0.021
Dyslipidaemia	4/30 (13.3%)	8/21 (38.1%)	0.051
Ischemic stroke	0/30 (0.0%)	3/21 (14.3%)	0.064
Donor age, years	38 [25–44]	42 [29–47]	0.393
Male donor sex	17/30 (56.7%)	11/17 (64.7%)	0.759
Donor BMI, kg/m^2^	24.2 [22.6–26.1]	24.5 [22.0–27.6]	0.674
Donor noradrenaline	20/28 (71.4%)	5/17 (29.4%)	0.012
Donor CRP, mg/dL	20.6 [6.5–110.7]	11.9 [4.0–26.2]	0.294
Donor amylase, U/L	31 [24–57]	32 [20–71]	0.919
Donor LDH, U/L	293 [242–522]	349 [198–508]	0.977
Donor PRDI	1.35 [0.93–1.64]	1.43 [0.95–1.73]	0.566
Total operating time, min	150 [120–180]	180 [120–194]	0.540
Cold ischemia time, min	330 [300–420]	360 [329–424]	0.516
Warm ischemia time, min	38 [30–45]	34 [30–40]	0.152
Intraoperative blood transfusion, units	0 [0–1]	0 [0–0]	0.194
ASA class	3 [2–3]	3 [2–3]	0.743

Continuous variables are median [IQR]. Categorical variables are n/N (%), excluding missing values from denominators. PRA CDC maximum was compared using a rank-based Mann–Whitney U test as a continuous variable.

**Table 3:** Spectral QC metrics by pancreas graft outcome group.

QC Metric	Overall (n = 51)	Preserved (n = 30)	Dysfunctional (n = 21)
Baseline drift index (% of abs. area)	5.28 ± 3.76	5.21 ± 3.25	5.39 ± 4.47
Cosine similarity vs. cohort median	0.905 [0.833–0.929]	0.902 [0.802–0.937]	0.906 [0.881–0.928]
Amide I SNR	31.8 [14.8–75.5]	33.8 [12.6–79.3]	28.5 [13.2–72.4]
Spike count (fingerprint, MAD |z| > 6)	63 [35.5–93]	66 [36–98.8]	58 [33–85]

Values are mean ± SD for approximately normal distributions; otherwise, median [IQR]. Normality was assessed per group (Shapiro–Wilk), and test choice followed the pre-specified rule: parametric testing only when both groups were consistent with normality.

**Table 4:** Performance of the principal models with confusion-matrix readout.

Workflow	AUC	Accuracy	Precision	Recall	Specificity	Correctly Classified
Clinical-only, FCBF	0.860	0.804	0.809	0.804	0.763	Dysfunction 14/21 Preserved 27/30
FTIR-only, 2nd derivative + vector normalization + locked FCBF-selected wavenumbers	0.997 (95% CI 0.985–1.000)	0.961 (95% CI 0.902–1.000)	0.964	0.961	0.973	Dysfunction 21/21 Preserved 28/30 Permutation AUC *p* = 0.001
FTIR + clinical	0.990	0.961	0.963	0.961	0.944	Dysfunction 19/21 Preserved 30/30
FTIR-only, Group 1 vs. Group 3	0.992	0.930	0.930	0.930	0.883	Group 1: 29/30 Group 3: 11/13

Abbreviations: AUC, area under the receiver-operating characteristic curve; CI, confidence interval; FCBF, Fast Correlation-Based Filter; FTIR, Fourier-transform infrared spectroscopy. Confidence intervals for the final FTIR-only model were estimated by stratified non-parametric bootstrap resampling of the LOOCV prediction set. For the Group 1 versus Group 3 analysis, Group 1 denotes preserved pancreas and preserved kidney graft function, whereas Group 3 denotes pancreas graft dysfunction with preserved kidney graft function.

**Table 5:** Selected features in the best-performing spectral and combined models.

Analysis	Selected Variables	Interpretation
Primary FTIR-only model	≈1301, ≈3033, ≈1224, ≈2960, ≈3113, ≈1098, ≈879, ≈843, ≈1119 cm^−1^	Discriminative spectral surrogates within the composite serum FTIR fingerprint; individual molecular assignments are tentative
FTIR + clinical model	PRA CDC maximum (%), ≈1301, ≈1224, ≈844, ≈3113, ≈3013, ≈1098 cm^−1^, ischemic stroke, dyslipidaemia	Combined clinical variables and discriminative serum spectral features; not interpretable as molecule-specific biomarkers
Group 1 vs. Group 3 FTIR model	≈1304, ≈844, ≈3165, ≈3100, ≈2809, ≈945, ≈1041, ≈1205, ≈2939 cm^−1^	Discriminative spectral pattern associated with pancreas dysfunction despite preserved kidney function

## Data Availability

The data presented in this study are available on request from the corresponding author. The data are not publicly available due to privacy and ethical restrictions.
